# Antiretroviral therapy during the neonatal period

**DOI:** 10.4102/sajhivmed.v16i1.361

**Published:** 2015-05-04

**Authors:** James J.C. Nuttall

**Affiliations:** 1 Red Cross War Memorial Children's Hospital, Cape Town, South Africa; 2 Department of Paediatrics and Child Health, University of Cape Town, South Africa

## Rationale for initiating combination antiretroviral therapy during the neonatal period

Initiation of combination antiretroviral therapy (cART) at 6–9 weeks of age has been shown to reduce early infant mortality by 76% and HIV progression by 75% compared with cART deferred until clinical or CD4 criteria were met.^1^ In the landmark Children with HIV Early Antiretroviral Therapy (CHER) trial, although the median age of starting cART in the early treatment arm was 7.4 weeks, one-third (10/30) of the overall mortality in the trial occurred in the early treatment arm.^1^ In another study, 62% of 403 infants who initiated cART at median 8.4 weeks of age already had advanced HIV disease (CD4 < 25% or < 1500 cells/mm^3^ or World Health Organization [WHO] Stage 3 or 4) at initiation.^2^

The above, with other findings describing the benefits of early cART, raise the question of whether even earlier cART initiation – immediately after birth or during the neonatal period – could further reduce morbidity and mortality rates, and confer greater benefits, particularly for infants who acquired HIV infection during the pregnancy and are consequently at highest risk of rapid disease progression.^3,4,5,6,7^ The potential of early neonatal cART initiation in modifying the longer-term trajectory of HIV infection in an individual patient and need for lifelong cART is an area of intensive research.

The recent shift to targeted HIV polymerase chain reaction (PCR) testing at birth rather than only at 6 weeks of age allows for the earliest detection of neonates in whom intrauterine transmission of HIV infection has occurred and has opened the door to neonatal cART initiation. Availability of validated point-of-care HIV PCR testing will further increase the drive to initiate cART during the early neonatal period.

Safety and efficacy data on neonatal cART is currently very limited. There is even less experience with treating premature and low birth weight neonates with cART. Uncertainties relate to pharmacokinetics (PK), dosing, safety and choice of cART regimen. In addition, timing of the transition from prophylactic antiretroviral (ARV) regimens aimed at prevention of transmission to cART regimens aimed at long-term treatment requires further investigation.

## Outline of pharmacokinetics, dosing and safety of antiretrovirals during the neonatal period

### Nucleoside reverse transcriptase inhibitors

#### Abacavir

Despite the South African (SA) ARV treatment guidelines recommendation that Abacavir (ABC) should be used in all first-line cART regimens for children, there are insufficient safety data to recommend the use of ABC in infants < 3 months old.^8^ There is also a lack of PK studies to guide dosing in this age group.^9^

#### Lamivudine

The SA ARV drug dosing chart (2013) recommends a Lamivudine (3TC) dose of 2 mL (20 mg) twice daily from 3 kg – 4.9 kg but advises expert consultation for neonates and infants weighing < 3 kg.^10^ Although 3TC is not Food and Drug Administration (FDA) approved for use in infants < 3 months of age, it has been used and studied in neonates. The recommended dose for neonates (< 4 weeks of age) for either prevention of transmission or treatment is 2 mg/kg/dose twice daily. The recommended paediatric dose (age ≥ 4 weeks) is 4 mg/kg/dose twice daily to a maximum dose of 150 mg twice daily.^11^ These recommendations are based on population PK analyses in infants < 6 weeks of age.^12,13^ The higher WHO dosage recommendations (3 mL [30 mg] twice daily from 3 kg – 4.9 kg) result in increased plasma concentrations compared with the 2 mg/kg/dose recommendations and should be avoided in neonates.^14,15^ There are no published data to guide dosing in premature neonates.

Lamivudine has generally been associated with minimal toxicity in older children and adults but studies suggest that haematological toxicity (anaemia, neutropaenia, thrombocytopaenia) increases when combined zidovudine (AZT)/3TC neonatal prophylaxis is used when compared with AZT alone, with increasing numbers of patients requiring treatment discontinuation or blood transfusions.^16^ Lamivudine may be given without regard to food, and the oral solution may be stored at room temperature. Excretion is via the renal route, and dose adjustment is required in renal insufficiency.^11^

#### Stavudine

The recommended dose for neonates from birth to 13 days of age is 0.5 mg/kg/dose twice daily and, from 14 days onwards, 1 mg/kg/dose twice daily to a maximum of 30 mg twice daily.^11,17^ Dose reduction is recommended if there is renal dysfunction. There are no published data to guide Stavudine (D4T) dosing in premature neonates.

The oral liquid formulation requires the addition of water to powder, has a concentration of 1 mg/mL, requires refrigeration and is stable for 30 days.^11^ An alternative dosing method using opened capsules (available as 15 mg, 20 mg or 30 mg) with the contents dispersed in a small amount of water and the appropriate dose administered via oral syringe, has been investigated and plasma exposure shown to be equivalent to ingested whole capsules.^18^ Stavudine is no longer included in SA treatment guidelines, and the oral liquid formulation is not readily available in the public sector.^8^

Although there is limited experience in the context of neonatal cART, D4T in older infants and children generally has minimal short-term toxicity and good efficacy.^11^ It may therefore be a consideration for short-term use in neonatal cART when AZT is contraindicated or haematological toxicity has occurred. Alternative options for substitution of AZT are limited owing to lack of ABC safety and dosing information in infants < 3 months of age.

#### Zidovudine

There is considerable experience with the use of AZT in the neonatal period, although mostly for prevention of transmission. Although the landmark PACTG 076 study of prevention of mother-to-child transmission of HIV (PMTCT) used dosing of 2 mg/kg/dose 6 hourly, more recent data support twice-daily dosing.^19^ Current USA guidelines recommend a dose of 4 mg/kg/dose twice daily for either prevention of transmission (4–6 weeks) or treatment (4 weeks) for neonates with gestational age ≥ 35 weeks.^11^

Western Cape (South Africa) PMTCT guidelines (2014) incorporate combination AZT/ Nevirapine (NVP) prophylaxis to prevent transmission in high-risk infants and recommend a standardised AZT dose according to birth weight (> 2 kg: 12 mg 12-hourly; < 2 kg: 4 mg/kg 12-hourly) or gestational age (< 35 weeks: 2 mg/kg 12-hourly) administered as post-exposure prophylaxis for 4 weeks.^20^

The SA ARV drug dosing chart (2013) and WHO weight band dosing (2010) recommend a dose of 6 mL (60 mg) twice daily from 3 kg – 5.9 kg which is equivalent to 10 mg – 20 mg/kg/dose or 172 mg – 300 mg/m^2^/dose but advises expert consultation for neonates and infants weighing < 3 kg.^10,15^

It is recognised that the standard paediatric AZT dose (240 mg/m^2^/dose twice daily) may lead to haematological toxicity (anaemia, neutropaenia, thrombocytopaenia), particularly in premature neonates with anaemia of prematurity and where other agents that may cause bone marrow suppression (e.g. ganciclovir, co-trimoxazole) are administered concurrently. Specific dosing based on PK studies in premature neonates is available and close monitoring of haematological parameters is recommended (at least every 2–4 weeks) during the neonatal and early infant period.^11,21^ Switching from AZT to an alternative medication should be considered if signs of haematological or other toxicity are severe or persistent. Dosing adjustment is required in the setting of renal insufficiency or hepatic impairment.^11^

An intravenous (IV) AZT formulation is available. It is generally used in the setting of prevention of transmission when the neonate is unable to tolerate oral medication.^11^ As no other IV ARV formulations are available, a fully IV treatment regimen is not feasible. Monotherapy with IV AZT as treatment in HIV-infected neonates is not recommended.

After 6 weeks of age, AZT dosing according to the SA ARV drug dosing chart (2013) is recommended.^10^ Alternatively, body surface area-based dosing (240 mg/m^2^/dose twice daily) may be used.^11^

### Non-nucleoside reverse transcriptase inhibitors

#### Nevirapine

Although the use of NVP is well established for prevention of transmission, the optimal treatment dose for neonates < 14 days of age has not been established. The dose of oral suspension approved for treatment in neonates > 15 days of age and children is 200 mg/m^2^/dose twice daily with a maximum dose of 200 mg (immediate-release formulation) twice daily.^11^

The standard practice of using a 14-day lead-in dose when initiating treatment with NVP in order to allow induction of cytochrome p450 metabolising enzymes and reduce occurrence of rash, can lead to subtherapeutic plasma NVP levels. This effect increases the risk of developing drug resistance and associated worse virological and clinical outcomes. Initiating cART with full-dose twice-daily NVP in black African children < 2 years of age without previous NVP exposure resulted in fewer subtherapeutic NVP levels than the 14-day dose escalation approach, and none of the children less than 2 years of age who received full-dose NVP developed rash.^22^

In HIV-infected neonates who are transitioning from once-daily NVP prophylaxis for prevention of transmission to cART containing NVP, it would seem appropriate to use full-dose NVP with careful clinical and laboratory monitoring from the start of cART because some degree of induction of cytochrome p450 metabolising enzymes is likely to have already occurred. Further studies that include both neonates exposed to NVP prophylaxis for prevention of transmission and those without previous NVP exposure are required to validate the safety, efficacy and feasibility of full-dose NVP initiation. Based on PK modelling, the International Maternal Pediatric Adolescent AIDS Clinical Trials (IMPAACT) P1115 study will investigate a NVP treatment dose of 6 mg/kg/dose twice daily with full-dose initiation in full-term neonates < 48 hours of age.^23^

Nevirapine toxicities include rash, hypersensitivity reactions, and hepatotoxicity. Close monitoring for rash and liver function test abnormalities is required. NVP should not be administered to patients with moderate or severe hepatic impairment. In addition, drug-drug interactions are common and concomitant medications should be carefully reviewed prior to initiating NVP.^11^

Efavirenz, etravirine and rilpivirine are not approved for use in neonates and are not recommended.^11^

### Protease inhibitors

#### Lopinavir/ritonavir

Lopinavir/ritonavir (LPV/r) co-formulated oral solution (Kaletra) was first approved by the USA's FDA in 2000 for the treatment of HIV-infected children ≥ 6 months old. In 2008, Kaletra oral solution was approved for use in children ≥ 14 days old.^24^ The recommended dose of oral solution is 300 mg/m^2^/dose twice daily.^11^

In January 2011, the FDA released a statement on Kaletra toxicity in neonates.^24^ Post-marketing cases of toxicity were reported to the FDA's Adverse Event Reporting System (AERS) in September 2010 and were attributable to LPV and/or the inactive ingredients propylene glycol and ethanol. Kaletra oral solution contains 152.7 mg/mL of propylene glycol (15.3% w/v) and has a high ethanol content (356 mg/mL or 42.4% v/v).

A search of the AERS database revealed 10 reported cases with adverse events that might have been related to LPV, propylene glycol or ethanol. All 10 patients were neonates, and 8 of the 10 were premature neonates. Cardiac toxicity occurred in 7 of the patients and included bradycardia, sinoatrial block, complete atrioventricular block, congestive cardiomyopathy, cardiac failure and cardiogenic shock. An elevated lactate level was documented in 2 cases. Neuromuscular toxicity in 3 neonates included hypotonia, abnormal electroencephalogram (EEG), altered state of consciousness, somnolence and asthenia. Acute renal failure was seen in 5 neonates and an increased serum creatinine was documented in 1. Four neonates developed hyperkalaemia. Respiratory complications occurred in 3 neonates and included respiratory failure, pulmonary haemorrhage, respiratory arrest, dyspnoea and wheezing. Gastrointestinal events in 5 neonates included vomiting, failure to thrive, abdominal distension and ulcerative colitis. One of the 10 neonates died.^24,25^

Eight of the 10 neonates received their first dose of Kaletra within the first 2 days of life. The onset of toxicity occurred within 1–6 days in 8 of the neonates. A full-term infant showed the first signs of toxicity 20 days after birth. After Kaletra was discontinued, 6 neonates recovered within 5 days.^24,25^ Transient asymptomatic elevation in 17-hydroxyprogesterone levels has been reported in term neonates treated at birth with LPV/r.^26^

As an appropriate dose in premature infants and neonates < 14 days of age is not known and the consequences of Kaletra toxicity in premature infants can be severe or possibly fatal, the FDA strongly recommends that Kaletra should be avoided in this age group:^11,24^

If in the judgment of the health care professional, the benefit of using Kaletra oral solution in babies to treat HIV infection immediately after birth outweighs the potential risks, then the neonate should be monitored closely for increases in serum osmolality and serum creatinine and for toxicity related to Kaletra oral solution. These toxicities include hyperosmolality with or without lactic acidosis, renal toxicity, CNS depression (including stupor, coma, and apnea), seizures, hypotonia, cardiac arrhythmias, ECG changes and hemolysis.^24^

The IMPAACT P1030 study evaluated PK, safety and efficacy of LPV/r 300 mg/m^2^/dose twice daily in combination with 2 nucleoside analogues in 10 HIV-infected infants with an age range of 3.6–5.9 weeks. Amongst 9 infants completing intensive PK evaluation on a median dose of 267 mg/m^2^/dose (range 246–305) twice daily, the LPV area-under-the curve (AUC) was significantly lower than that in the 6-weeks to 6-months of age cohort. The LPV trough level (C_min_) ranged from 0.99 µg/mL – 4.87 µg/mL which did not differ from the older children. A plasma LPV trough concentration of ≥ 1 µg/mL has been used as a correlate of efficacy in treatment-naïve adults. After 24 weeks’ follow-up, adverse events were limited to transient neutropaenia in 3 infants, and only 2 of 10 infants met criteria for protocol-defined virological failure. Noting the low LPV exposure (AUC), the authors advised careful dose calculation and frequent dose adjustment for weight gain, and proposed studying a higher dose for very young infants.^27,28^

Taking into account the increasing usage of LPV/r administration to neonates at risk of perinatal or postnatal HIV transmission, Urien et al.^29^ aimed to determine optimal dosing for LPV/r in the first weeks of life based on a population PK approach incorporating body weight, gestational age and postnatal age in 96 infants from France and the UK. Amongst the 96 neonates, 7 were on treatment for neonatal HIV infection. The median postnatal age was 2 weeks (range 1 day – 102 weeks), median gestational age was 38 weeks (range 27.3–41 weeks), median body weight was 3.3 kg (range 1.16 kg – 10.4 kg). A total of 163 LPV concentrations were available for analysis. The mean LPV dosage was 590 mg/m^2^/day (range 106–1454) or 39 mg/kg/day (range 11–110) dosed between 1 and 3 times per day. A one-compartment model described the data with body weight and age being the main influential covariates. The following dosage regimen was derived from the predicted LPV trough concentrations using a therapeutic range of 1 µg/mL – 8 µg/mL: 40 mg 12-hourly, 80 mg 12-hourly and 120 mg 12-hourly for 1 kg – 2 kg, 2 kg – 6 kg and 6 kg – 10 kg groups respectively.^29^

Holgate et al.^30^ described the use of Kaletra-based cART in 8 HIV-infected premature neonates treated between 2006 and 2011 in Cape Town. The median gestational age at birth was 31 weeks (range 27–33), median age at initiation of LPV/r-based cART was 26.5 days (range 5–96), median corrected gestational age at cART initiation was 34.1 (range 31.6–44.7) and median dose of LPV/r at time of measuring LPV levels was 287 mg/m^2^/dose or 23.1 mg/kg (range 235–325; 21.1–28.6, respectively). LPV trough levels were sampled a median 7 days after LPV/r initiation, 3 infants had subtherapeutic plasma levels (< 1 µg/mL), and in 1 infant the plasma level was above the recommended target range (> 4 µg/mL). The dose of LPV/r was adjusted in the 3 infants with low levels and a median dose of 533 mg/m^2^ (range 400–540) resulted in plasma levels within the recommended therapeutic range. Overall, 5/8 infants required doses > 300 mg/m^2^ to achieve plasma LPV trough levels within the recommended range. No adverse effects attributable to LPV/r solution were observed. The study highlighted the role of therapeutic drug monitoring in order to achieve target trough LPV levels. Although no toxicity was observed, the authors emphasised the need for extreme caution and careful monitoring of premature neonates treated with Kaletra.^30^

Although nelfinavir (NFV) has been used in ART regimens for prevention of transmission and treatment, it is not currently recommended for treatment in children < 2 years of age as there is significant inter-individual variation in plasma levels and insufficient PK data to support a standardised dosing regimen.^11^

Atazanavir, darunavir, fosamprenavir, indinavir, full-dose ritonavir (RTV), saquinavir and tipranavir are not approved for use in neonates and are not recommended.^11^

### Integrase inhibitors

#### Raltegravir

There are currently no published data on safety and dosing of Raltegravir (RAL) in neonates. However, Phase 1 and 2 studies are underway. The IMPAACT P1097 study investigated washout PK in neonates born to HIV-infected mothers and showed that the neonatal half-life of RAL varied between 9 and 184 hours, most likely owing to reduced capacity for metabolism and elimination in newborns.^31^ The Phase 1 IMPAACT P1110 trial is investigating the safety and PK of RAL suspension (granules for suspension) in HIV-exposed neonates at high risk of acquiring HIV infection. Different dosing strategies between birth and 6 weeks of age will be investigated with the option of continuing RAL beyond 6 weeks of age in infants found to be HIV-infected.^32^

The IMPAACT P1066 study investigated the use of RAL oral suspension in combination with an optimised background ARV regimen in 26 HIV-infected infants and young children aged 4 weeks to 2 years who had previously received ARV medication for prevention of perinatal transmission. Clinical outcomes were acceptable, there were no treatment discontinuations owing to adverse events, and PK parameters were similar to those achieved amongst cohorts of older children in the same study.^33^ Raltegravir oral suspension is currently approved by the FDA for use in infants > 4 weeks of age and > 3 kg body weight.^11^ In SA, RAL suspension is not currently registered by the Medicines Control Council. In the SA public sector, RAL has been restricted for use in third-line ART regimens.

Dolutegravir and elvitegravir are not approved for use in neonates and are not recommended.^11^

## Choice of combination antiretroviral therapy regimen in neonates

Current SA HIV treatment guidelines recommend ABC+3TC+LPV/r as first-line cART for all HIV-infected children < 3 years of age; there are no separate recommendations for full-term or premature neonates.^8^ There are currently no published clinical trial data comparing different cART regimens initiated during the neonatal period. Clinical trials in older infants and young children have informed treatment recommendations in these age groups and are important to consider in relation to cART initiation during the neonatal period.

The IMPAACT P1060 trial showed that LPV/r-based cART was virologically superior to NVP-based cART both in infants with previous exposure to NVP (6–36 months of age) and in infants without previous exposure to NVP (3–36 months of age).^34,35^ One of the reasons proposed to explain the difference in virological efficacy between NVP and LPV/r is the dose escalation strategy used when initiating NVP, as this has been associated with subtherapeutic plasma NVP levels which could increase the risk of developing drug resistance and treatment failure.^22^ Initiation of cART with full-dose NVP has been investigated and further studies are underway.^22,23^

An additional possible explanation for the difference in outcomes relates to the presence of drug resistance mutations prior to cART initiation. In settings of high nucleoside reverse transcriptase inhibitor (NRTI) exposure owing to the use of NVP for prevention of transmission and use of NRTI/ Non-nucleoside reverse transcriptase inhibitor (NNRTI) regimens during pregnancy, the presence of resistance mutations may compromise the efficacy of NVP-based regimens in the neonate. HIV drug resistance mutations in plasma virus were determined using population sequencing in 230 newly-diagnosed HIV-infected children < 2 years of age in Johannesburg in 2011. Two-thirds of the HIV-infected children had been exposed to maternal and/or infant PMTCT, 56.8% had NNRTI, 14.8% NRTI and 1.3% PI mutations. In children with no reported PMTCT exposure, resistance to NNRTI was detected in 24%, to NRTI in 10.7% and to PI in 1.3%. In the children who were tested at ≤ 8 weeks of age, 85% had NNRTI drug resistant mutations present.^36^

The above data support the use of PI-based first-line cART in HIV-infected infants and young children regardless of PMTCT history. However, owing to potentially severe and life-threatening short-term toxicity concerns, Kaletra is currently not recommended in neonates < 14 days old and < 42 weeks corrected gestational age. Long-term toxicities of PIs, including effects on growth and lipid metabolism, have also been reported.^37,38^

Other studies have investigated whether 4 rather than 3 ARV drugs could improve outcomes based on the observation that young infants commonly have very high HIV viral loads and the hypothesis that increased regimen potency might achieve more rapid virological suppression, immunological recovery and better long-term treatment efficacy. However, the available studies are heterogeneous in nature and therefore difficult to compare directly, and 3 drug regimens have remained the standard-of-care in most guidelines.

The PACTG 356 study showed superior virological suppression rates of a 4-drug regimen comprising D4T/3TC/NVP/NFV compared with either AZT/3TC/ABC/NVP or AZT/3TC/NVP.^39^ Prendergast et al.^40^ showed that infants randomised to receive immediate or deferred (until CD4 count reached < 20%) 4-drug cART comprising AZT/3TC/NFV/NVP were able to achieve excellent adherence and virological suppression after one year by intention-to-treat analysis of 80%. The European Pregnancy and Pediatric HIV Cohort Collaboration (EPPICC) observational study of 437 infants initiating cART during the first year of life showed better virological and immunological responses among those starting 4-drug NNRTI-based regimens than 3-drug NNRTI-based and LPV/r-based regimens after median 5.9 years of follow-up.^41^ The AntiRetroviral Research for Watoto (ARROW) trial conducted among 3-month – 17-year-old children in Uganda and Zimbabwe showed no long-term (72 weeks) immunological benefit to starting 4-drug cART (NNRTI +3 NRTIs) then simplifying to 3-drug cART (either NNRTI + 2 NRTIs, or 3 NRTIs) compared with starting 3-drug cART (NNRTI + 2 NRTIs), and there was increased toxicity with 4-drug cART.^42^

Further clinical trials comparing safety, tolerability and efficacy of different cART regimens, including consideration of 4-drug and triple class regimens, initiated during the neonatal period in high burden settings are warranted in order to better guide treatment recommendations.

## Transition from antiretroviral prophylaxis to treatment

Neonatal ARV prophylaxis regimens vary between guidelines. In SA, prophylaxis regimens include NVP alone, AZT alone, NVP + AZT, and NVP + AZT + 3TC, and recommended duration ranges from 6 weeks to 3 months or more.^8,43^ As a result, most neonates will be receiving ARV prophylaxis at the time that a positive birth HIV PCR test result is obtained.

**FIGURE 1:**
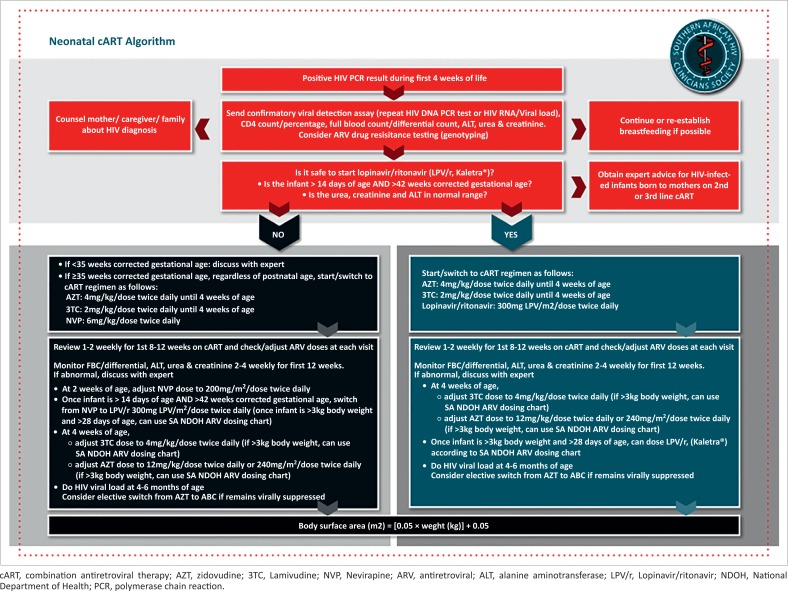
Recommended process for initiation of combination antiretroviral therapy in neonates.

Transition from neonatal ARV prophylaxis to neonatal cART requires adjustment to the number and choice of ARVs, dosage and dose frequency in most cases. Clinical assessment of the neonate, baseline investigations and careful counselling of the mother and family are pre-requisites to cART initiation. Although optimal strategies for transition from prophylaxis to cART have not been widely studied, it is recommended that a standardised approach applicable to the majority of HIV-infected neonates is adopted (Figure 1). Expert opinion and individualised guidance will still be required for certain categories of neonates; for example, low birth weight, premature or unwell neonates.

After obtaining a positive HIV PCR test result in a neonate, the following actions are required prior to consideration of cART initiation:

- A blood sample for confirmatory viral detection assay (second HIV DNA PCR test as per current National Department of Health [NDOH] SA guidelines or HIV RNA/viral load) must be submitted to the laboratory.^8,43^ Initiation of cART should not be delayed on the basis that the result of the confirmatory PCR test has not yet been obtained. Rapid and systematic follow-up of birth HIV PCR tests that might have been submitted to the laboratory by the birthing facility or a referring clinic or hospital, and recall of HIV-positive neonates, is essential.
- The clinical condition of the neonate must be assessed. This includes determining the corrected gestational age (in weeks), postnatal age, birth weight and current weight, presence of any vital organ dysfunction including neonatal jaundice, hepatitis or renal dysfunction, and presence of other congenital or acquired infections including syphilis, tuberculosis (TB) and cytomegalovirus as indicated by the maternal history and clinical state of the neonate. Co-morbidities and their treatment may alter the timing of cART initiation and the treatment regimen required in the neonate. In neonates who are clinically unstable at the time that HIV infection is diagnosed, ARV prophylaxis should be discontinued and the neonate stabilised and treated as necessary prior to initiation of cART. In addition, neonates who are not fully established on enteral feeding are not eligible to initiate cART.
  ■ HIV-infected neonates who are also exposed to and/or infected with TB require evaluation as to the infectiousness and drug sensitivity profile of the contact, and assessment (clinical, radiological, bacteriological) of TB infection/disease followed by anti-TB chemoprophylaxis or treatment. HIV-infected neonates initiated on rifampicin and receiving LPV/r-based cART will require additional RTV (0.75 x LPV dose) to be added to the cART regimen although PK, safety and efficacy data for the super-boosting strategy in the neonatal age group is lacking. The PK, safety and efficacy of rifampicin in combination with NVP-based cART in the neonatal age group is also not known. Expert advice should be obtained.
- Baseline blood investigations should be performed including full blood count and differential white cell count, urea and creatinine, and alanine aminotransferase (ALT).
- Careful and detailed counselling of the mother and if possible other family members who will be involved in the care of the neonate is required. The mother of the child might not have disclosed her own HIV status to other family members, and disclosure of the neonate's HIV status to the family should be discussed. In addition to providing support, counselling should include information about the HIV diagnosis in the neonate as well as details about cART. Information and guidance on infant feeding should be provided. Mothers who had chosen to breastfeed should be encouraged to continue breastfeeding whilst mothers who had chosen formula feeding should consider switching to breastfeeding if feasible.
- Ideally, a blood sample should be submitted for ARV drug resistance testing (genotyping) prior to initiation of cART in the neonate. This is particularly relevant when the neonate has been exposed to maternal cART or ARV prophylaxis prior to the diagnosis of HIV infection, and may assist in determining optimal ARV drug choices in future cART regimens for the child. Expert advice should be obtained to assist in the management of HIV-infected neonates born to mothers on 2nd- or 3rd-line cART regimens.

In neonates ≥ 15 days of age and ≥ 42 weeks corrected gestational age and with normal renal and hepatic function, an initial regimen of AZT+3TC+LPV/r is recommended. Refer to Table 1 and Figure 1 for guidance on dosing and monitoring.

**TABLE 1:** Antiretroviral drugs, formulations and dose recommendations for treatment of full-term neonates.

Inhibitor	ARV drug	Formulations	Dose	Comment
Nucleoside reverse transcriptase Inhibitors	Abacavir	20 mg/mL	Neonatal dose not known	Not FDA approved for infants < 3 months of age
	Lamivudine	10 mg/mL	Birth – 4 weeks of age: 2 mg/kg/dose twice daily ≥ 4 weeks of age: If < 3 kg body weight: 4 mg/kg/dose twice daily If ≥ 3 kg body weight: may be dosed according to SA ARV dosing chart	Not FDA approved for infants < 3 months of age but generally well tolerated. May contribute to haematological toxicity
	Stavudine	Oral suspension: powder for reconstitution with water 1 mg/mL; Capsules: 15 mg, 20 mg, 30 mg	Birth – 13 days of age: 0.5 mg/kg/dose twice daily ≥ 14 days of age: 1 mg/kg/dose twice daily	Consider use if AZT contraindicated or haematological toxicity Reconstituted oral suspension: requires refrigeration, stable for 30 days, no longer readily available Capsules may be opened, contents dispersed in water and appropriate dose administered
	Zidovudine	10 mg/mL	Birth – < 4 weeks of age (≥ 35 weeks gestational age): 4 mg/kg/dose twice daily ≥ 4 weeks of age: If < 3 kg body weight: 12 mg/kg/dose twice daily or 240 mg/m^2^/dose twice daily If ≥ 3 kg body weight: may be dosed according to SA ARV dosing chart	Monitor for haematological toxicity
Non-nucleoside reverse transcriptase inhibitor	Nevirapine	10 mg/mL	≤ 14 days of age: treatment dose is undetermined Investigational dose (IMPAACT P1115): 6 mg/kg/dose twice daily ≥ 15 days of age: If < 3 kg body weight: 200 mg/m^2^/dose twice daily If ≥ 3 kg body weight: may be dosed according to SA ARV dosing chart	Monitor for rash, hypersensitivity reactions, hepatotoxicity
Protease inhibitor	Lopinavir/ ritonavir (Kaletra)	80 mg/20 mg LPV/r per 1 mL	300 mg/m^2^/dose twice daily Doses may require adjustment based on therapeutic drug monitoring, if available	Contraindicated < 14 days of age and < 42 weeks corrected gestational age Refer to text for details on monitoring required

*Source*: Adapted from Ref. 11

Note: Body surface area (m^2^) = (0.05 x weight [kg]) + 0.05.

ARV, antiretroviral; LPV/r, Lopinavir/ritonavir; SA ARV, South African antiretroviral; IMPAACT, International Maternal Pediatric Adolescent AIDS Clinical Trials; FDA, Food and Drug Administration; AZT, zidovudine.

In full-term neonates ≤ 14 days of age, approved dosing recommendations are only available for AZT and 3TC. Treatment with only 2 drugs is not recommended. Based on a detailed review of available PK data, an NVP dose of 6 mg/kg/dose twice daily in full-term neonates initiating cART < 48 hours of age is under investigation in the IMPAACT P1115 study. Pending the results of this and other studies, a provisional recommendation to use an initial regimen of AZT+3TC+NVP dosed twice daily in neonates ≥ 35 weeks gestational age at birth, regardless of postnatal age, with normal hepatic function and appropriate monitoring for toxicity seems reasonable. Refer to Table 1 and Figure 1 for guidance on dosing and monitoring. As there are data for older infants and young children showing superior efficacy of LPV/r-based, compared with NNRTI-based, cART, these neonates should be considered for elective switch to AZT+3TC+LPV/r when they are ≥ 15 days of age and ≥ 42 weeks corrected gestational age.

The decision to initiate cART in premature neonates < 35 weeks gestational age involves assessing the relative risks and benefits of using unapproved dosing and the inherent concerns regarding drug toxicity. Dosing recommendations for premature neonates are currently only available for AZT. Treatment with AZT alone is not recommended and, based on currently available data, it is recommended that Kaletra should be avoided in this age group (refer to Table 2). If cART is initiated in premature neonates, expert guidance on dosing and toxicity monitoring should be obtained.

**TABLE 2:** Antiretroviral drugs, formulations and dose recommendations for treatment of premature neonates.

Inhibitor	ARV drug	Formulations	Dose	Comment
Nucleoside reverse transcriptase inhibitors	Abacavir	20 mg/mL	Appropriate dose for treatment of premature neonates is not known	Not FDA approved for infants < 3 months of age
	Lamivudine	10 mg/mL	Appropriate dose for treatment of premature neonates is not known	Not FDA approved for infants < 3 months of age but generally well tolerated. May contribute to haematological toxicity
	Stavudine	Oral suspension: powder for reconstitution with water 1 mg/mL; Capsules: 15 mg, 20 mg, 30 mg	Appropriate dose for treatment of premature neonates is not known	Reconstituted oral suspension: requires refrigeration, stable for 30 days, no longer readily available Capsules may be opened, contents dispersed in water and appropriate dose administered
	Zidovudine	10 mg/mL	≥ 35 weeks gestation: birth – < 4 weeks of age: 4 mg/kg/dose twice daily ≥ 4 weeks of age: If < 3 kg body weight: 12 mg/kg/dose twice daily or 240 mg/m2/dose twice daily If ≥ 3 kg body weight: may be dosed according to SA ARV dosing chart ≥ 30 to < 35 weeks gestation: birth – < 2 weeks of age: 2 mg/kg/dose twice daily ≥ 2 weeks – < 8 weeks of age: 3 mg/kg/dose twice daily ≥ 8 weeks of age: 12 mg/kg/dose twice daily < 30 weeks gestation: birth – < 4 weeks of age: 2 mg/kg/ dose twice daily ≥ 4 weeks – < 10 weeks: 3 mg/kg/dose twice daily ≥ 10 weeks of age: 12 mg/kg/dose twice daily	Monitor for haematological toxicity
Non-nucleoside reverse transcriptase inhibitor	Nevirapine	10 mg/mL	Appropriate dose for treatment of premature neonates is not known	Monitor for rash, hypersensitivity reactions, hepatotoxicity
Protease inhibitor	Lopinavir/ritonavir (Kaletra)	80 mg/20 mg LPV/r per 1 mL	Appropriate dose for treatment of premature neonates is not known	Contraindicated < 14 days of age and < 42 weeks corrected gestational age. Refer to text for details on monitoring required

*Source*: Adapted from Ref. 11

Note: Zidovudine (AZT) is the only ARV drug for which dosing for treatment of premature neonates is approved. However, treatment with AZT monotherapy is not recommended. Body surface area (m^2^) = (0.05 x weight [kg]) + 0.05.

ARV, antiretroviral; LPV/r, Lopinavir/ritonavir; FDA, Food and Drug Administration.

cART, combination antiretroviral therapy; AZT, zidovudine; 3TC, Lamivudine; NVP, Nevirapine; ARV, antiretroviral; ALT, alanine aminotransferase; LPV/r, Lopinavir/ritonavir; NDOH, National Department of Health; PCR, polymerase chain reaction.
